# Short-term effects of rainfall on childhood hand, foot and mouth disease and related spatial heterogeneity: evidence from 143 cities in mainland China

**DOI:** 10.1186/s12889-020-09633-1

**Published:** 2020-10-09

**Authors:** Fan Yang, Yue Ma, Fengfeng Liu, Xing Zhao, Chaonan Fan, Yifan Hu, Kuiru Hu, Zhaorui Chang, Xiong Xiao

**Affiliations:** 1 Department of Epidemiology and Biostatistics, West China School of Public Health and West China Fourth Hospital, Sichuan University, No. 16, Section 3, South Renmin Road, Chengdu, Sichuan 610041 PR China; 2 Division of Infectious Disease & Key Laboratory of Surveillance and Early Warning on Infectious Disease, Chinese Center for Disease Control and Prevention, 155 Changbai Road, Changping District, Beijing, 102206 PR China; 3 Institute of Basic Medical College, Chinese Academy of Medical Sciences, Beijing, China

**Keywords:** Childhood hand, foot and mouth disease, Rainfall, Exposure-response relationship, Multicity time series analysis, Spatial heterogeneity

## Abstract

**Background:**

Numerous studies have demonstrated the potential association between rainfall and hand, foot and mouth disease (HFMD), but the results are inconsistent. This study aimed to quantify the relationship between rainfall and HFMD based on a multicity study and explore the potential sources of spatial heterogeneity.

**Methods:**

We retrieved the daily counts of childhood HFMD and the meteorological variables of the 143 cities in mainland China between 2009 and 2014. A common time series regression model was applied to quantify the association between rainfall and HFMD for each of the 143 cities. Then, we adopted the meta-regression model to pool the city-specific estimates and explore the sources of heterogeneity by incorporating city-specific characteristics.

**Results:**

The overall pooled estimation suggested a nonlinear exposure-response relationship between rainfall and HFMD. Once rainfall exceeded 15 mm, the HFMD risk stopped increasing linearly and began to plateau with the excessive risk ratio (ERR) peaking at 21 mm of rainfall (ERR = 3.46, 95% CI: 2.05, 4.88). We also found significant heterogeneity in the rainfall-HFMD relationships (*I*^*2*^ = 52.75%, *P* < 0.001). By incorporating the city-specific characteristics into the meta-regression model, temperature and student density can explain a substantial proportion of spatial heterogeneity with *I*^*2*^ statistics that decreased by 5.29 and 6.80% at most, respectively.

**Conclusions:**

Our findings verified the nonlinear association between rainfall and HFMD. The rainfall-HFMD relationship also varies depending on locations. Therefore, the estimation of the rain-HFMD relationship of one location should not be generalized to another location.

## Background

Hand, foot and mouth disease (HFMD) is an acute highly contagious viral infection caused by enteroviruses and predominantly affects children under 5 years of age [1, 2]. While symptoms for most children is generally mild, few cases may experience more severe symptoms involving the central nervous system, like acute flaccid paralysis, encephalitis, meningitis, and even fatal cardiopulmonary failure [3]. As a result of continuously large outbreaks and high rates of severe cases, East and Southeast Asia represent the worst hit areas around the world in terms of HFMD epidemics [4–7]. In mainland China, HFMD is the leading childhood infection among all notifiable diseases and responsible for millions of clinical cases [8] and greater than 75,000 age-weighted disability adjusted life years (DALYs) annually [9].

Multiple studies suggested that the water environment plays a crucial role in the survival of enterovirus (i.e., the pathogen of HFMD), thereby potentially affecting the transmission of HFMD. Water environments not only provide the major natural reservoir for enterovirus [10] but also protect enterovirus from diverse factors resulting in inactivation, such as extreme temperature, ultraviolet radiation, and salinity [11, 12]. In addition, the water environment can facilitate the attachment of enterovirus to suspended particles, which further prolongs enterovirus survival time [13]. As the main source of natural water environments, rainfall was thus considered as an important driving factor of HFMD epidemics.

Although a growing number of epidemiological studies have been recently conducted to quantify the associations between rainfall and HFMD, evidence from previous studies remains inconsistent. Studies in both southern China [14] and Thailand [15] reported that the HFMD incidence was positively associated with rainfall. In contrast, studies in Hong Kong, Singapore, Japan, Taiwan [16], and Korea [17] suggested a nonsignificant association between rainfall and HFMD. A study in Vietnam [18] even suggested a negative association. The inconsistency of results among studies may originate from multiple sources. First, the diversity of methodologies and model choices adopted in different studies would inevitably affect the results. In fact, the same data could generate dissimilar conclusions when subject to different analyses [19]. Second, the potential nonlinear exposure-response relationship could be another explanation for the inconsistencies [20, 21] because the estimated associations between rainfall and HFMD may vary depending on the level of rainfall. Finally, the rainfall-HFMD relationship could also be modified by location-specific characteristics [22, 23]. To date, the spatial heterogeneity of rainfall-HFMD relationships across studies remains poorly understood, thus hindering the understanding of potential mechanisms and the usage of rainfall information to predict HFMD epidemics.

To address the above research gap, we proposed a multicity two-stage time series study including 143 cities in mainland China. By estimating the associations between rainfall and HFMD for each of the 143 cities under a common modeling framework, we aimed to assess the spatial heterogeneity of rainfall-HFMD relationships and further explore the potential reasons.

## Methods

### Data sources

Details of data used in this study have been described previously [22, 23]. Briefly, we retrieved surveillance data of HFMD cases and meteorological variables between from 1 January 2009 to 31 December 2014 in mainland China. We excluded data before 2009 due to the presence of less reliable data [24], and data after 2014 were excluded to reduce the potential influence of vaccines on the natural history of HFMD [25]. More specifically, surveillance data of HFMD cases were collected from the China Information System for Disease Control and Prevention. Here, HFMD cases were defined as patients with vesicular or papular rashes on hands, feet, mouth or buttocks with or without fever [26]. We further focused our analysis on children aged 0–12 years (accounted for greater than 99% of cases) to allow our results to be more clearly interpretable. Daily surveillance data of meteorological variables were collected from the China Meteorological Data Sharing System based on national ground meteorological stations. Meteorological variables included accumulated rainfall (from 20:00 to 20:00), mean relative humidity, mean temperature, sunshine hours, and mean air pressure.

Given that most of HFMD cases came from urban areas, we aggregated and matched the daily time series of HFMD and meteorological variables of prefectural-level cities (corresponds to medium to large size cities under China’s administrative structure) in mainland China. Confining our analysis to prefectural-level cities also assures better data quality. Finally, we included 143 cities with successful matching in our final analysis. To further explore the sources of spatial heterogeneity, city-specific characteristics were collected from the China city statistical yearbook [27], including economic characteristics (GDP per person and GDP growth rate), demographic characteristics (population density, population growth rate and student density), health resources (hospital beds and licensed physicians) and traffic (total travel passengers). To represent the climatic differences among cities, the arithmetic means of daily meteorological variables were used for each of the 143 cities as proxy variables.

### Statistical analysis

#### Study design

We adopted a multicity two-stage time series design to robustly estimate rainfall-HFMD relationships and explore potential heterogeneity [19]. In the first stage, we implemented a common time series regression model [28] for each of the 143 cities and obtained the city-specific estimations of rainfall-HFMD relationships. In the second stage, we pooled the city-specific rainfall-HFMD relationships using meta-analysis and examined whether city-specific characteristics can explain the spatial heterogeneity [29].

#### First-stage analysis

In summary, we applied a common quasi-Poisson time series regression with parametric splines to quantify the associations between rainfall and HFMD for each of the 143 cities. We used the quasi-Poisson distribution was assumed to account for overdispersion of disease counts. The natural cubic spline with 8 degrees of freedom (df) per year was used to control for the seasonality and long-term trends (i.e., time-varying unmeasured confounding) [22]. We also included the indicators of weekdays and holidays and exponentially weighted moving averages of other meteorological variables (temperature, sunshine and air pressure) with the exception of relative humidity to remove residual confounding variables. Humidity was excluded because humidity and rainfall are highly correlated [30]. Given that HFMD is an infectious disease, we also incorporated the autoregressive term of HFMD daily counts at lag 1 and 2 days in logarithmic form to match the transmission mechanism, which was informed by the classical Susceptible-Infectious-Recovery (SIR) model [31].

In previous studies, rainfall was incorporated into models as either a continuous [32, 33] or categorical variable [34]. For fair comparisons with previous studies, we adopted rainfall as both continuous and categorical variables in this study, whereas the other model choices were identical. For the continuous scale, we applied the natural cubic spline with 5 df to characterize the potential non-linear exposure-response relationship between rainfall and HFMD, which was determined by varying a series of df (see more details in Additional file 3). For the categorical scale, we divided the daily accumulated rainfall into four levels based on the overall percentiles, including the reference group (rainfall ≤10th percentile or 0.07 mm), low rainfall group (rainfall between 10th and 50th percentiles or 0.07 ~ 1.35 mm), high rainfall group (rainfall between 50th and 90th percentiles or 1.35 ~ 7.10 mm) and extreme rainfall group (rainfall >90th percentiles or 7.10 mm) [14, 35]. Then, rainfall was incorporated as a dummy variable with delayed effects in the above time series regression. For both approaches, the same lag range between 0 to 14 lag days and exponentially weighted moving averages were used to characterize the delayed effects based on previous studies [32, 36, 37].

#### Second-stage analysis

Corresponding to the two approaches used in the first-stage analysis, we applied different methods to obtain the overall rainfall-HFMD relationships depending on the variable types of rainfall. For continuous-scale rainfall, city-specific nonlinear exposure-response relationships between rainfall and HFMD were pooled by the multivariate meta-regression model with random intercepts [29] to obtain the overall exposure-response curve. For categorical-scale rainfall, city-specific estimates corresponding to different levels of rainfall (i.e., low, high and extreme rainfall versus reference) were pooled by three separate univariate meta-regression models with random intercepts [38] to obtain the overall estimates of each level of rainfall, separately. We also measured and tested the spatial heterogeneity of city-specific rainfall-HFMD relationships using *I*^*2*^ statistics and the Cochran *Q* test [38].

To further explore the reasons for potential heterogeneity, we incorporated the city-specific characteristics as meta-predictors into the above models with random intercepts. We included city-specific characteristics separately rather simultaneously (denoted as single predictor model) given that our main goal was to confirm potential effect modifiers rather than model predictions. Another added benefit of the single predictor model was the avoidance of collinearity issues due to high correlations among city-specific characteristics. Of note, the rainfall distribution was highly right skewed with sparse data in the high value range, which might lead to non-robust estimations of the right tail of exposure-response curves. Therefore, we only reported the results of meta-predictors for the categorical scale rainfall in the main text. Additional details of the corresponding results for continuous-scale rainfall are provided in Additional file 1 and Additional file 2, and these results were consistent with our main findings.

#### Sensitivity analysis

To exclude the possibility that the model choices may substantially affect our conclusions, we implemented additional sensitivity analysis regarding key model choices in the time series regression models. The key model choices included the df of the natural cubic splines used to characterize the rainfall-HFMD relationships, the lag range of the rainfall-HFMD relationships and alternative cut-off points of rainfall groups (5th, 50th, and 95th percentiles). Additional details of our sensitivity analysis results are reported in Additional file 3, Additional file 4 and Additional file 5. In general, our main conclusions were quite robust for numerous model choices.

Statistical analyses were performed using R software (version 3.5.3), specifically *dlnm*, *mvmeta* and *metaphor* packages. Geographic maps were generated using ArcGIS Pro software (version 2.4, authorization number: ESU934769606).

## Results

**Fig. 1:**
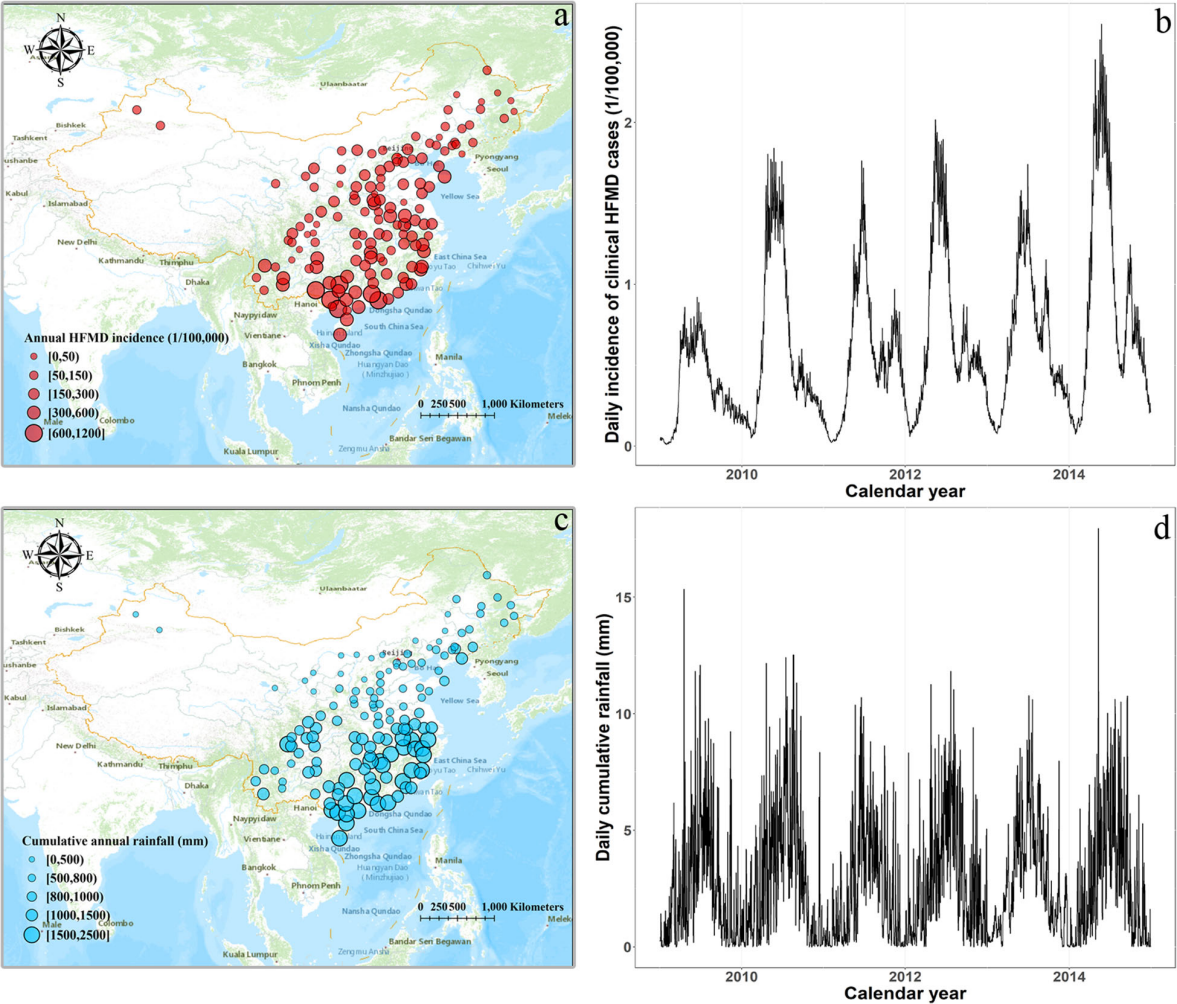
Spatial and temporal distributions of clinical HFMD incidences and cumulative rainfall for the 143 cities in mainland China from 2009 to 2014 (Source of map: ArcGIS Pro software). **a** Spatial distribution of annual clinical HFMD incidences. **b** Temporal distribution of daily incidences of clinical HFMD cases. **c** Spatial distribution of annual cumulative rainfall. **d** Temporal distribution of daily cumulative rainfall. For the sub-figure b and d, we used the average mean of daily incidence of HFMD and daily cumulative rainfall among the 143 cities for the sake of simplicity. All the map sources were provided by the ArcGIS Pro software

From 2009 to 2014, by including 143 cities in mainland China, we retrieved a total of 3,200,157 HFMD clinical cases in children under 12 years old. Our study area covered the majority of mainland Chinese with great diversity in geographic and climatic conditions. We found that HFMD circulated widely throughout the whole country. In general, HFMD incidences steadily increased from north to south. We particularly observed a high incidence area in the southernmost part of China where the annual HFMD incidences for multiple cities exceeded 1000 cases per 100,000 persons (Fig. 1a). Similarly, the spatial distribution of annual rainfall exhibited an increasing trend from north to south. The whole southern and southeastern coast of China experienced high rainfall levels (Fig. 1c). Regarding temporal trends, we observed an annual major peak in the time series of HFMD incidences, which typically occurred from May to August (Fig. 1b). The annual peak of rainfall roughly echoed that of HFMD incidence (Fig. 1d). Given that similar spatial-temporal patterns were observed between rainfall and HFMD, it was reasonable to assume that rainfall might be an environmental driver of the HFMD epidemic.

**Fig. 2:**
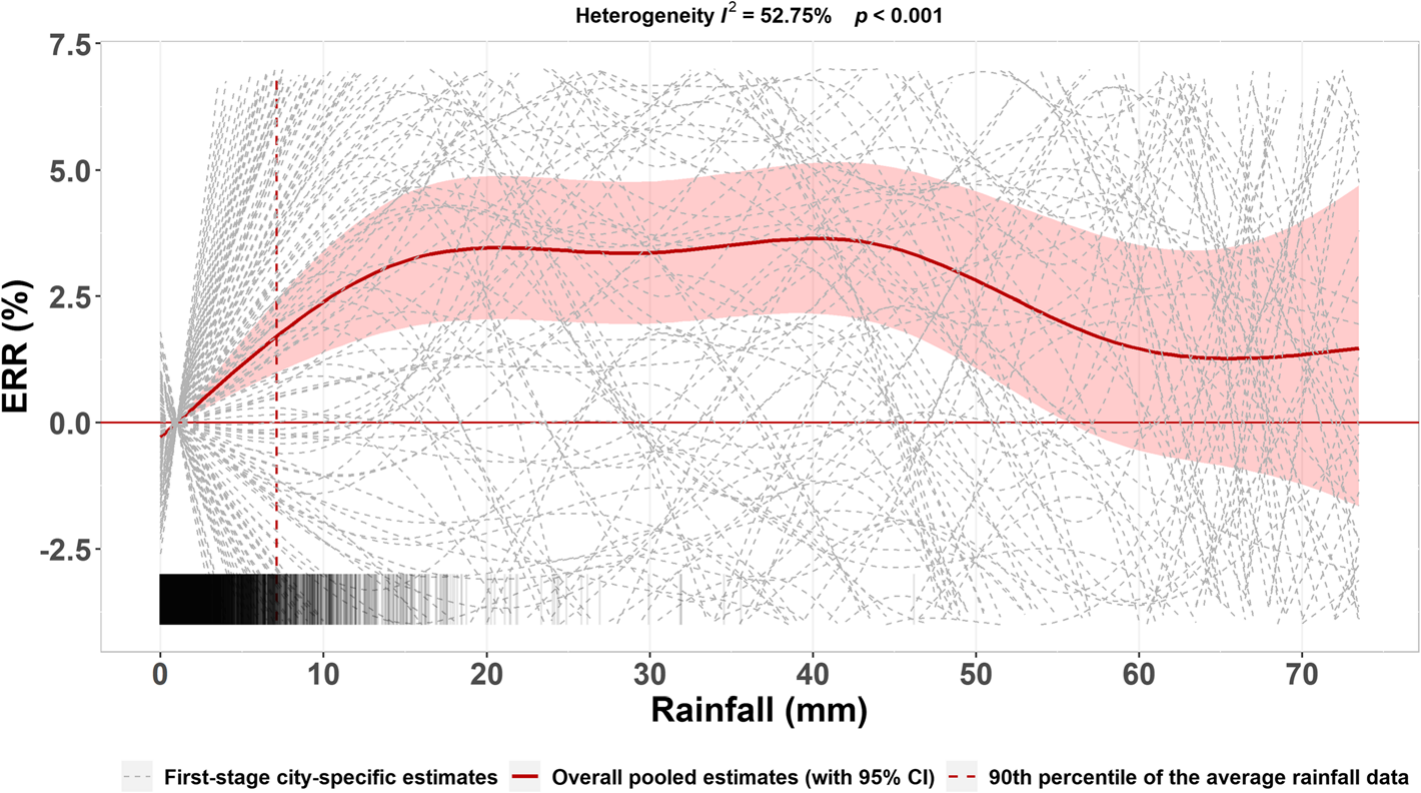
City-specific and overall pooled estimates of exposure-response curves between rainfall and HFMD. Stripes in the bottom represented the density of the rainfall data. ERR represents the excessive risk ratio (ERR, %), which was calculated by the transformation of risk ratio, i.e., (RR-1) × 100%

Based on continuous-scale rainfall data, we first pooled the city-specific estimates of exposure-response curves between rainfall and HFMD based on the multivariate meta-regression model (Fig. 2). The overall pooled estimates suggested a marginal effect on the rainfall-HFMD relationship with substantial heterogeneity among cities (*I*^*2*^ statistics = 52.75%, *P* < 0.001). We observed an approximately linear increase in the HFMD risk as rainfall initially increased. Once rainfall exceeded 15 mm, the HFMD risk began to plateau and was even reduced as rainfall further increased. However, we would prefer not to overinterpret the results of extreme rainfalls due to limited data. Overall, we observed a nonlinear association between rainfall and HFMD, and the excessive risk ratio peaked at 21-mm rainfall (ERR = 3.46, 95% CI: 2.05, 4.88).

**Fig. 3:**
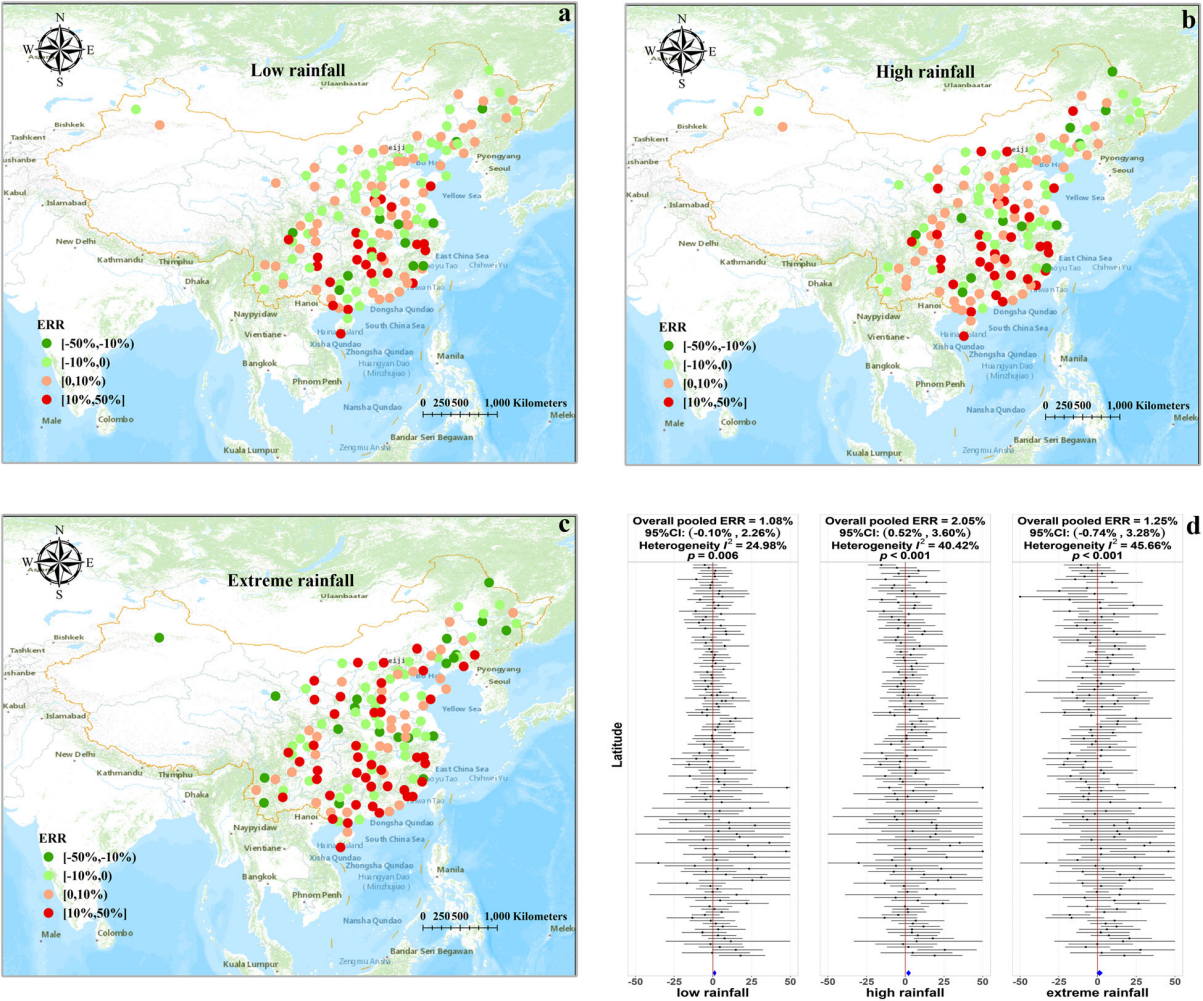
City-specific and overall pooled estimates of rainfall-HFMD associations for different rainfall levels (Source of map: ArcGIS Pro software). **a** Spatial distribution of rainfall-HFMD associations in the low rainfall group. **b** Spatial distribution of rainfall-HFMD associations in the high rainfall group. **b** Spatial distribution of rainfall-HFMD associations in the extreme rainfall group. **d** Forest plot of city-specific estimates (measured by ERR) ordered by latitudes for different rainfall levels. Overall pooled estimates were represented by the blue diamond at the bottom, and we truncated the range of ERR from − 50 to 50 to save space. All the map sources were provided by the ArcGIS Pro software

For categorical-scale rainfall, city-specific estimates for different levels of rainfall were pooled separately by univariate meta-regression model (Fig. 3). The results were consistent with those obtained for continuous-scale rainfall. Compared to the reference group, the overall pooled estimate (measured by ERR) of low, high and extreme rainfall were 1.08% (95% CI: − 0.10, 2.26%), 2.05% (95% CI: 0.52, 3.60%) and 1.25% (95% CI: − 0.74, 3.28%), respectively (Fig. 3d). A steady increase in rainfall-HFMD associations were noted from the low to high rainfall group, whereas no further increase was observed in the extreme rainfall group. Based on the spatial distribution of city-specific estimates, we found that all groups showed similar spatial variations in rainfall-HFMD associations, and the highest values were clustered in south-central China (Fig. 3a-c). Similar to continuous-scale rainfall results, substantial heterogeneity was observed in categorical-scale rainfall results. In addition, an upward trend of heterogeneity was noted, and the *I*^*2*^ statistics increased from 24.98% in the low rainfall group to 45.66% in the extreme rainfall group.

**Fig. 4:**
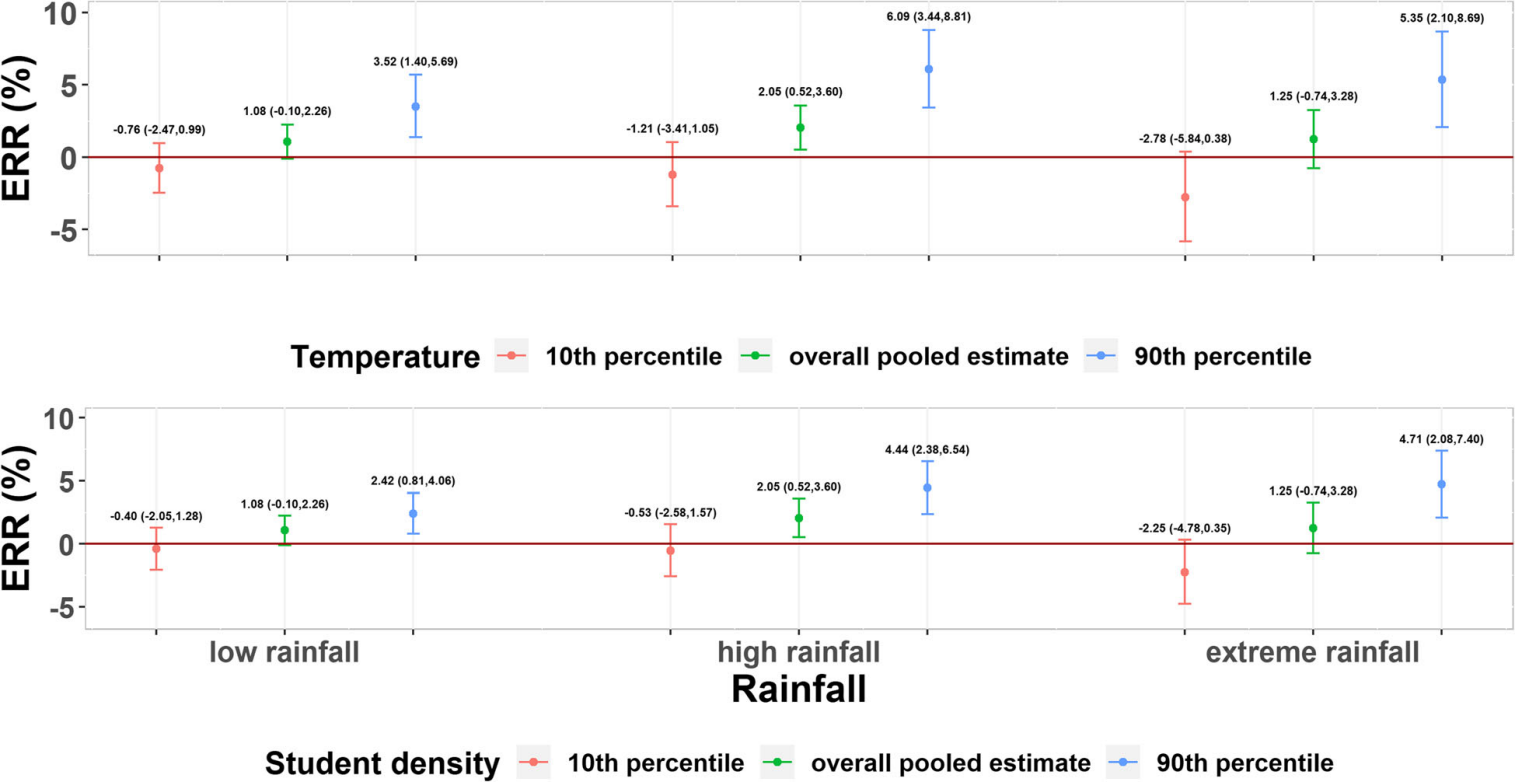
The predicted rainfall-HFMD relationship in ERR for the 10th and 90th percentiles of temperature and student density

We further examined the potential reasons for spatial heterogeneity in the rainfall-HFMD associations using the univariate meta regression which incorporates city-specific characteristics as explanatory variables. We found that temperature and student density could explain a substantial portion of heterogeneity regardless of rainfall groups. Both variables could reinforce the associations between rainfall and HFMD (Table 1). In the low and high rainfall groups, temperature explained the largest proportion of heterogeneity, and the *I*^*2*^ statistics decreased by 3.23 and 5.29%, respectively (Table 1). Compared to 10th percentile of temperature, the estimated ERR of the 90th percentile of temperature increased from − 0.76% (95% CI: − 2.47, 0.99%) to 3.52% (95% CI: 1.40, 5.69%) and from − 1.21% (95% CI: − 3.41, 1.05%) to 6.09% (95% CI: 3.44, 8.81%) in the low and high rainfall groups, respectively (Fig. 4). However, in the extreme rainfall group, student density explained a slightly larger proportion of heterogeneity compared with temperature, and the *I*^*2*^ statistics decreased by 6.80% (Table 1). Compared to the 10th percentile of student density, the estimated ERR of the 90th percentile of student density increased from − 2.25% (95% CI: − 4.78, 0.35%) to 4.71% (95% CI: 2.08, 7.40%) (Fig. 4).

**Table 1:** Univariate meta-regression models by incorporating city-specific characteristics to explain heterogeneity

Meta-predictors	Low rainfall				High rainfall				Extreme rainfall			
	*β*^a^	*P*_*W*_^b^	*I*^*2*^ (%)	*P*_*Q*_^c^	*β*^a^	*P*_*W*_^b^	*I*^*2*^ (%)	*P*_*Q*_^c^	*β*^a^	*P*_*W*_^b^	*I*^*2*^ (%)	*P*_*Q*_^c^
Intercept only	–	–	24.98	0.006	–	–	40.42	< 0.001	–	–	45.66	< 0.001
Climatic variables												
Temperature	0.016 (0.004, 0.028)	0.007	21.75	0.019	0.027 (0.013, 0.042)	< 0.001	35.13	< 0.001	0.030 (0.011, 0.050)	0.002	41.80	< 0.001
Relative humidity	0.006 (− 0.006, 0.017)	0.320	25.66	0.006	0.012 (− 0.003, 0.026)	0.118	40.15	< 0.001	0.010 (− 0.011, 0.030)	0.348	45.66	< 0.001
Sunshine	−0.008 (− 0.020, 0.004)	0.195	24.95	0.007	−0.012 (− 0.028, 0.004)	0.136	40.29	< 0.001	− 0.010 (− 0.031, 0.011)	0.348	46.05	< 0.001
Air pressure	0.003 (−0.007, 0.013)	0.546	25.24	0.006	0.005 (−0.009, 0.019)	0.448	40.49	< 0.001	0.006 (−0.014, 0.026)	0.567	45.69	< 0.001
Demographic variables												
Student density	0.013 (0.002, 0.024)	0.018	22.26	0.016	0.023 (0.009, 0.036)	0.001	35.33	< 0.001	0.032 (0.016, 0.049)	< 0.001	38.86	< 0.001
Population density	0.004 (−0.005, 0.014)	0.362	24.91	0.007	0.010 (−0.003, 0.022)	0.131	39.61	< 0.001	0.013 (−0.004, 0.031)	0.130	44.82	< 0.001
Population increase	−0.002 (− 0.014, 0.009)	0.686	25.39	0.005	0.007 (−0.008, 0.023)	0.339	40.27	< 0.001	0.003 (−0.016, 0.023)	0.723	46.00	< 0.001
Health resources												
Hospital beds	0.009 (−0.003, 0.022)	0.153	23.72	0.009	0.012 (−0.004, 0.029)	0.129	39.96	< 0.001	0.015 (−0.006, 0.036)	0.155	45.17	< 0.001
Licensed physicians	0.006 (−0.005, 0.017)	0.304	25.45	0.006	0.010 (−0.004, 0.025)	0.158	40.49	< 0.001	0.014 (−0.004, 0.033)	0.127	45.10	< 0.001
Economic variables												
GDP per person	0.005 (−0.006, 0.015)	0.351	25.07	0.006	0.008 (−0.006, 0.022)	0.246	40.21	< 0.001	0.024 (0.005, 0.042)	0.011	42.13	< 0.001
GDP increase	0.003 (−0.009, 0.015)	0.600	25.63	0.005	0.000 (−0.015, 0.015)	0.999	40.72	< 0.001	−0.001 (− 0.021, 0.020)	0.951	45.89	< 0.001
Traffic	0.001 (−0.008, 0.010)	0.862	25.50	0.005	0.004 (−0.008, 0.017)	0.507	40.59	< 0.001	0.010 (−0.006, 0.026)	0.207	45.05	< 0.001

^a^ *β* is the coefficient of each meta-predictor, which was obtained using the restricted maximum-likelihood method

^b^ *P*_*W*_ is the *P*-value of the Wald test, which was used to test the significance of the coefficient of each meta-predictor

^c^ *P*_*Q*_ is the *P*-value of the Cochran *Q* test, which was used to test the significance of the residual heterogeneity

## Discussion

To our knowledge, this is the first nation-wide study to examine rainfall- HFMD relationships and related heterogeneity. Based on the two-stage time series analysis consisting of 143 cities in mainland China, we observed a significant positive association between rainfall and HFMD overall. This finding was in consistent with the earlier hypothesis that rainfall facilitates enterovirus survival by providing a natural water environment and increasing the humidity of the air, which subsequently increase HFMD risk [39, 40].

However, it is worth noting that the strength of the relationship between rainfall and HFMD was much weaker than that of temperature and humidity reported in similar nation-wide studies [22, 23]. Unlike temperature and humidity, rainfall can be characterized with a spike at zero (i.e., its values at most days were zero), which could further cause the estimation of exposure-response relationship to progress toward a null value [41]. In addition, rainfall also reduces host outdoor activity and effective contacts between susceptible and infectious individuals [42], which may subsequently hinder the spread of HFMD infection. Both of the above mechanisms could help to explain why many previous studies report a nonsignificant association between rainfall and HFMD and the relatively weaker estimation of an exposure-response relationship reported in this study.

Interestingly, we identified a nonlinear association between rainfall and HFMD. More specifically, we found that the HFMD risk stopped increasingly linearly and began to plateau once rainfall exceeded 15 mm. Our findings were consistent with other studies conducted in Singapore [43], south China [20, 32, 44] and Hong Kong [45], which also suggested that these associations would decrease with high levels of rainfall. We hypothesized that two forces drive the nonlinear behavior of the rainfall-HFMD relationship in opposite directions. Given the low level of rainfall, we hypothesized that the impacts of rainfall on host activity would be negligible; thus, the main impact of rainfall is facilitating enterovirus survival and increasing the HFMD risk. Nevertheless, given the high level of rainfall, we hypothesized that the main impact of rainfall would subsequently change to reducing host activity and hindering the spread of HFMD. These findings implied that weather could affect the HFMD risk via multiple pathways and complicated mechanisms.

After implementing a common time series model, we still observed significant spatial heterogeneity in the rainfall-HFMD relationships among different cities. In addition, we found that the heterogeneity of city-specific estimates increased as rainfall increased. As stated earlier, the distribution of rainfall data is highly right skewed with most data clustered in the low rainfall groups. Thus, estimates in the high rainfall lack statistical power and exhibit larger variations compared with the low rainfall group, which may subsequently explain the increased heterogeneity in the high rainfall group [46].

Furthermore, we found that temperature and student density explain a substantial portion of heterogeneity regardless of the rainfall level. Infectious disease epidemics are mainly determined by a sufficient number of susceptible and infectious individuals as well as effective contacts between susceptible and infectious individuals [47]. Regarding temperature, previous studies suggested that warm temperatures can simultaneously promote the excretion of enterovirus [48] and host activity [42], which can increase the concentration of infectious agents in the environment and effective contacts, respectively. Regarding student density, given that children are most vulnerable to the HFMD infections [1], high student density reflects a high proportion of susceptible individuals in the population, which can directly raise the effective contacts between susceptible and infectious individuals [49, 50]. Both abovementioned mechanisms contribute to the spread of HFMD, thereby reinforcing the associations between rainfall and HFMD. The robust results among different levels of rainfall further verified our hypothesis.

Unlike most previous studies that typically applied a single site analysis, this study included 143 cites in mainland China with great variations in climate, economic development, and health resources. The large sample size and significant diversity allow us to robustly estimate the rainfall-HFMD relationship and thoroughly examine potential heterogeneity. However, several limitations should also be noted and improved in further studies. First, given the time series study design, this study is vulnerable to the issue of ecological fallacies [51]. Although this study may be weak in terms of causal reference, the associations identified in this study can still provide important information on public health practices, like the weather-based early warning system for contagious disease. Second, a large portion of heterogeneity remained unexplained despite the incorporation of a wide range of city-specific characteristics into the meta-regression model. Limited by data accessibility, some other important city-specific characteristics should be taken into consideration in future studies, such as public health interventions, dominant subtypes of enterovirus, vegetation coverage, and soil features. Third, for the sake of simplicity, we did not take the spatial correlation into account when pooling city-specific estimates [28]. While the spatial correlation can be eliminated through incorporating city-specific characteristics into the meta-regression models, we believe that the impacts of spatial correlation on our conclusions should be negligible.

## Conclusions

In conclusion, this study verified the nonlinear association between rainfall and HFMD. These findings can help us to elucidate the complicated mechanisms by which rainfall is related to HFMD and provide insights into the establishment of weather-based early warning systems. Especially in the context of global climate change, the distribution of rainfall might experience substantial changes in the near future [52]. In addition, we also found that the rainfall-HFMD relationship is profoundly affected by local conditions. Therefore, the estimation of the rain-HFMD relationship of one location should not be generalized to another location.

## Supplementary information

**Additional file 1: Table S1.** Multivariate meta-regression models by incorporating city-specific characteristics to explain heterogeneity.

**Additional file 2: Figure S1.** The predicted rainfall-HFMD relationship in ERR for the 10th and 90th percentiles of temperature and student density.

**Additional file 3: Figure S2.** The overall pooled estimates of HFMD-rainfall relationships for different choice of df (the df of natural cubic splines used to characterized the exposure-response relationship).

**Additional file 4: Figure S3.** The overall pooled estimates of HFMD-rainfall relationships for different choice of lag range with the max lag time ranged from 7 to 21 days.

**Additional file 5: Table S2.** The pooled estimates (measured by ERR) of low, high and extreme rainfall with alternative cut-off points (5th, 50th, 95th percentile as cut-off points).

## Data Availability

The HFMD surveillance data are not publicly available, but access to this data could require permission from the Chinese Center for Disease Control and Prevention or from the corresponding author on reasonable request. The meteorological monitoring data and city-specific characteristics are publicly available, which can be obtained from the China Meteorological Data Sharing System (http://data.cma.cn) and the China city statistical yearbook respectively.

## Abbreviations

HFMD: Hand, foot and mouth disease
DALYs: Age-weighted disability adjusted life years
ERR: Excessive risk ratio
SIR: Susceptible-Infectious-Recovery
