# Supplementary material for: Short-term effects of rainfall on childhood hand, foot and mouth disease and related spatial heterogeneity: evidence from 143 cities in mainland China
Source: BMC Public Health. 2020 Oct 9;20:1528. doi: 10.1186/s12889-020-09633-1 (PMC7545871; doi:10.1186/s12889-020-09633-1)
Supplement: Supplementary file 1 — Additional file 1: Table S1. Multivariate meta-regression models by incorporating city-specific characteristics to explain heterogeneity. [file 12889_2020_9633_MOESM1_ESM.docx]

Table 1S Multivariate meta-regression models by incorporating city-specific characteristics to explain heterogeneity.

| **Meta-predictors** | Cochran Q test^a^ | | |  | LR test^b^ | | |  | *I2* |
| --- | --- | --- | --- | --- | --- | --- | --- | --- | --- |
|  | Stat | df | *p* |  | Stat | df | *p* |  | (%) |
| Intercept only | 1502.6 | 710 | <0.001 |  | — | — | — |  | 52.75 |
| Climatic variables | | | | | | | | | |
| Temperature | 1445.4 | 705 | <0.001 |  | 20.81 | 5 | 0.001 |  | 51.23 |
| Relative humidity | 1482.3 | 705 | <0.001 |  | 8.18 | 5 | 0.147 |  | 52.44 |
| Sunshine | 1475.9 | 705 | <0.001 |  | 8.96 | 5 | 0.110 |  | 52.23 |
| Air pressure | 1490.3 | 705 | <0.001 |  | 6.62 | 5 | 0.250 |  | 52.69 |
| Demographic variables | | | | | | | | | |
| Student density | 1460.8 | 705 | <0.001 |  | 11.23 | 5 | 0.047 |  | 51.74 |
| Population density | 1478.6 | 705 | <0.001 |  | 8.92 | 5 | 0.112 |  | 52.32 |
| Population increase | 1485.3 | 705 | <0.001 |  | 7.49 | 5 | 0.187 |  | 52.53 |
| Health resources | | | | | | | | | |
| Hospital beds | 1471.7 | 705 | <0.001 |  | 19.40 | 5 | 0.002 |  | 52.10 |
| Licensed physicians | 1466.9 | 705 | <0.001 |  | 17.07 | 5 | 0.004 |  | 51.94 |
| Economic variables | | | | | | | | | |
| GDP per person | 1469.0 | 705 | <0.001 |  | 9.79 | 5 | 0.081 |  | 52.01 |
| GDP increase | 1480.7 | 705 | <0.001 |  | 12.20 | 5 | 0.032 |  | 52.39 |
| Traffic | 1477.6 | 705 | <0.001 |  | 7.66 | 5 | 0.176 |  | 52.29 |

^a^ Cochran Q test was used to test the significance of residual heterogeneity with the null hypothesis as no heterogeneity.

^b^ LR test was used to test the significance of meta-predictors with the Intercept-only model as reference.
