# Supplementary figures and images for: Short-term effects of rainfall on childhood hand, foot and mouth disease and related spatial heterogeneity: evidence from 143 cities in mainland China
Source: BMC Public Health. 2020 Oct 9;20:1528. doi: 10.1186/s12889-020-09633-1 (PMC7545871; doi:10.1186/s12889-020-09633-1)

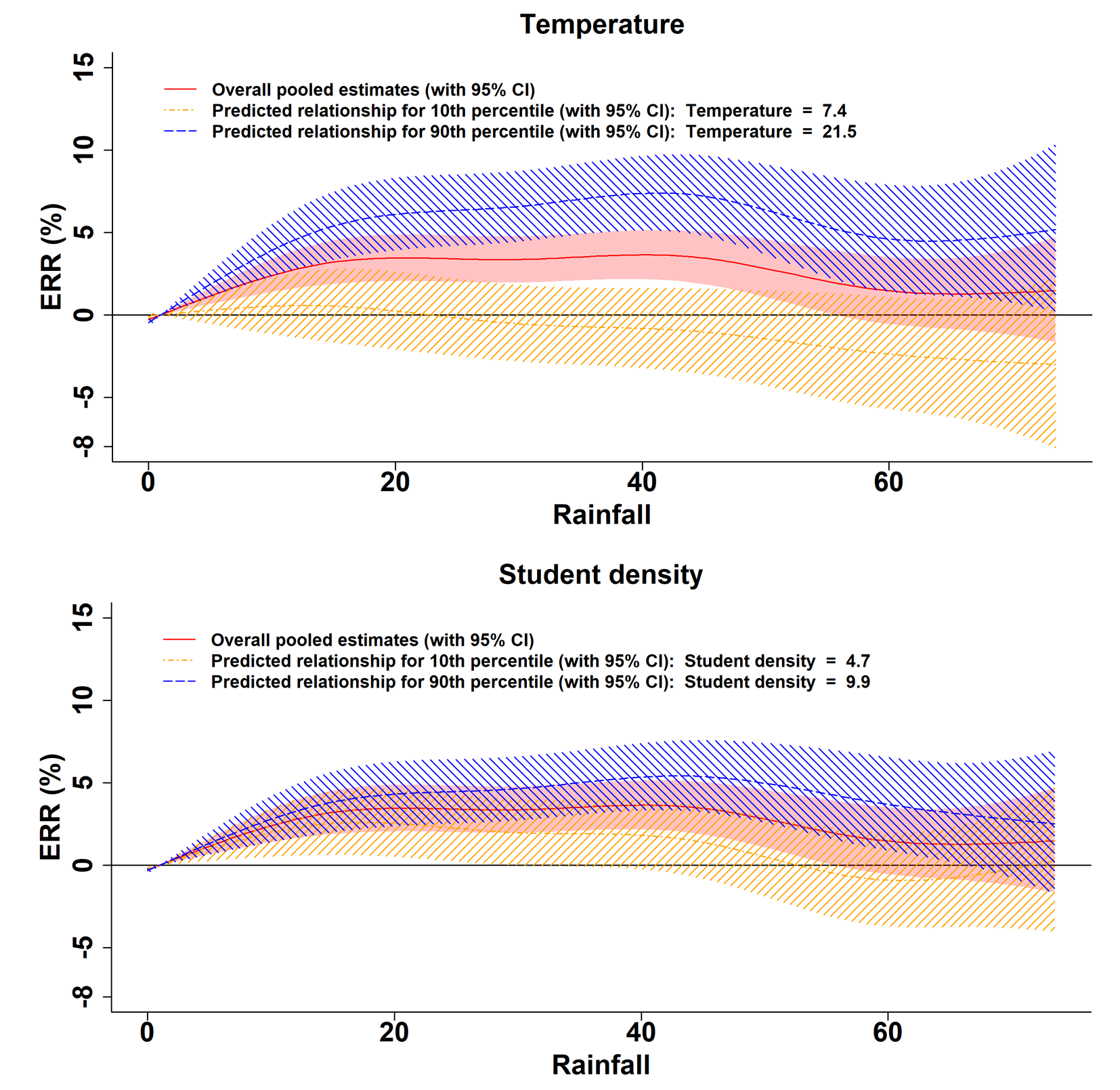

Supplement: Supplementary file 2 — Additional file 2: Figure S1. The predicted rainfall-HFMD relationship in ERR for the 10th and 90th percentiles of temperature and student density. [file 12889_2020_9633_MOESM2_ESM.tif]

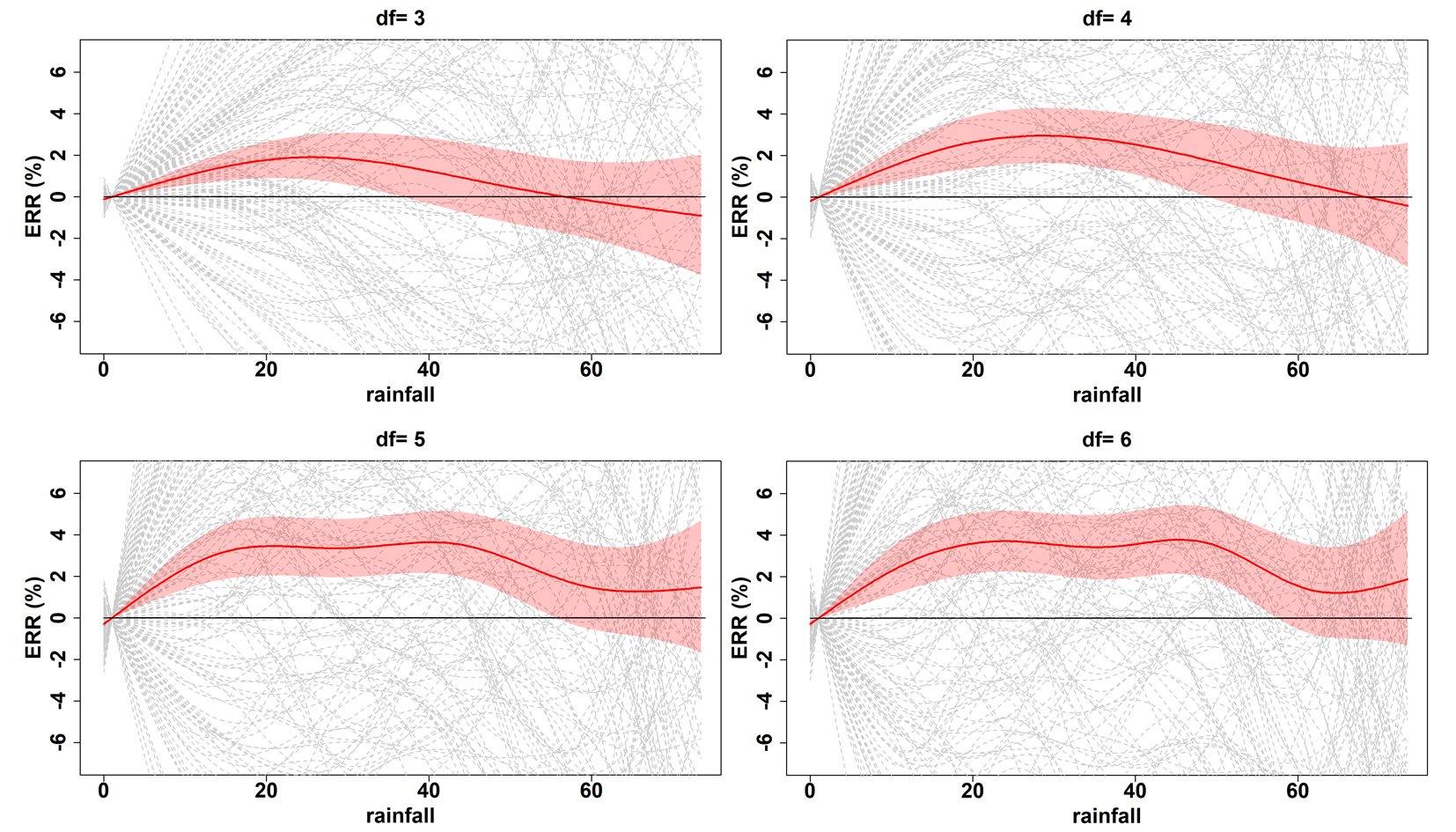

Supplement: Supplementary file 3 — Additional file 3: Figure S2. The overall pooled estimates of HFMD-rainfall relationships for different choice of df (the df of natural cubic splines used to characterized the exposure-response relationship). [file 12889_2020_9633_MOESM3_ESM.tif]

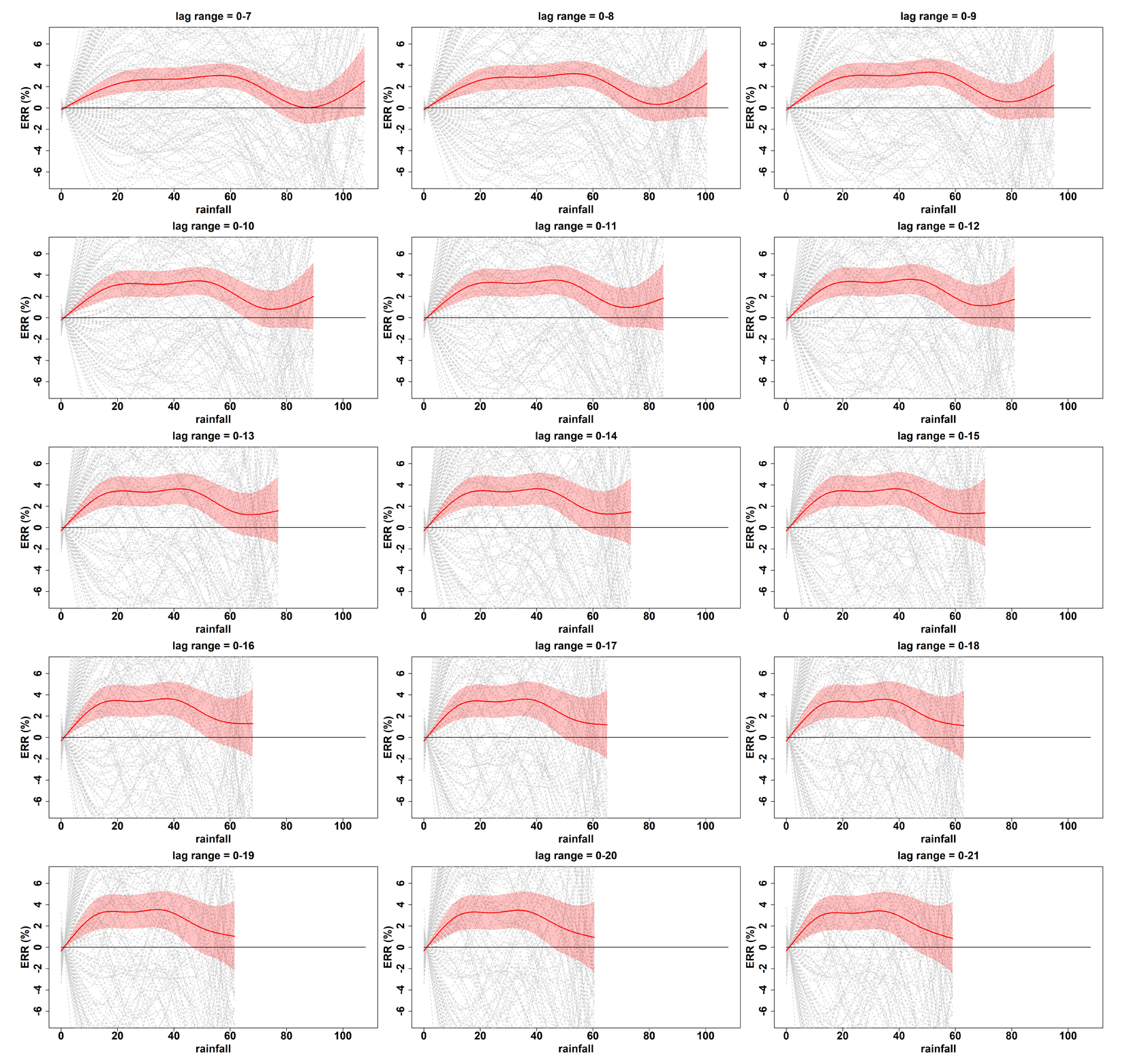

Supplement: Supplementary file 4 — Additional file 4: Figure S3. The overall pooled estimates of HFMD-rainfall relationships for different choice of lag range with the max lag time ranged from 7 to 21 days. [file 12889_2020_9633_MOESM4_ESM.tif]
