# Supplementary material for: Short-term effects of rainfall on childhood hand, foot and mouth disease and related spatial heterogeneity: evidence from 143 cities in mainland China
Source: BMC Public Health. 2020 Oct 9;20:1528. doi: 10.1186/s12889-020-09633-1 (PMC7545871; doi:10.1186/s12889-020-09633-1)
Supplement: Supplementary file 5 — Additional file 5: Table S2. The pooled estimates (measured by ERR) of low, high and extreme rainfall with alternative cut-off points (5th, 50th, 95th percentile as cut-off points). [file 12889_2020_9633_MOESM5_ESM.docx]

Table 2S The pooled estimates (measured by ERR) of low, high and extreme rainfall with alternative cut-off points (5^th^, 50^th^, 95^th^ percentile as cut-off points).

| ERR (%) | low rainfall | high rainfall | extreme rainfall |
| --- | --- | --- | --- |
| low bound | 0.40 | 0.72 | -0.87 |
| estimate | 1.97 | 2.66 | 1.78 |
| up bound | 3.57 | 4.63 | 4.51 |
